# MCC950 ameliorates ventricular arrhythmia vulnerability induced by heart failure

**DOI:** 10.1080/21655979.2022.2053813

**Published:** 2022-03-24

**Authors:** Xiaobo Jiang, Fan Yang, Dengke Ou, Luyong Huang, Hongfei Li, Mingjian Lang

**Affiliations:** a Department of Cardiology, Fifth People’s Hospital of Chengdu, Chengdu, PR China; b Department of Cardiology, Chengdu University of Traditional Chinese Medicine Affiliated Fifth People’s Hospital, Chengdu, China; c Department of Endocrinology, Fifth People’s Hospital of Chengdu, Chengdu, China; d Department of endocrinology, Chengdu University of Traditional Chinese Medicine Affiliated Fifth People's Hospital, Chengdu, China

**Keywords:** MCC950, NLRP3 inflammasome, ventricular arrhythmias, heart failure

## Abstract

MCC950, a specific NACHT, LRR, and PYD domains-containing protein 3 (NLRP3) inhibitor, has been reported to play a role in various cardiovascular diseases. However, its role in heart failure (HF)-induced ventricular arrhythmias (VAs) remains unclear. Hence, the present study aimed to clarify the role and underlying mechanisms of MCC950 in HF-induced VAs. Male C57BL/6 mice were induced with HF via transverse aortic constriction (TAC). Histological analysis, echocardiography, electrophysiological investigation, and western blot analysis were conducted to evaluate VA vulnerability induced by TAC and the potential mechanisms underlying the effects. MCC950 markedly improved cardiac function and decreased pulmonary edema induced by HF. Moreover, MCC950 also decreased VA vulnerability, as shown by the shortened QTc duration and action potential duration 90 (APD_90_), reduced APD alternans threshold, and decreased VA induction rate. Furthermore, MCC950 treatment significantly reversed TAC-induced cardiac hypertrophy and fibrosis. In addition, MCC950 administration increased the protein levels of ion channels (Kv4.2, KChIP2, and Cav1.2). Mechanistically, the above changes induced by MCC950 were due to the inhibition of the NLRP3 inflammasome. As a specific NLRP3 inhibitor, MCC950 significantly decreased HF-induced VA vulnerability by reversing cardiac structural remodeling and electrical remodeling, and the mechanism through which MCC950 exhibited this effect was inhibition of NLRP3 inflammasome activation.

## Graphical Abstract

**Figure:**
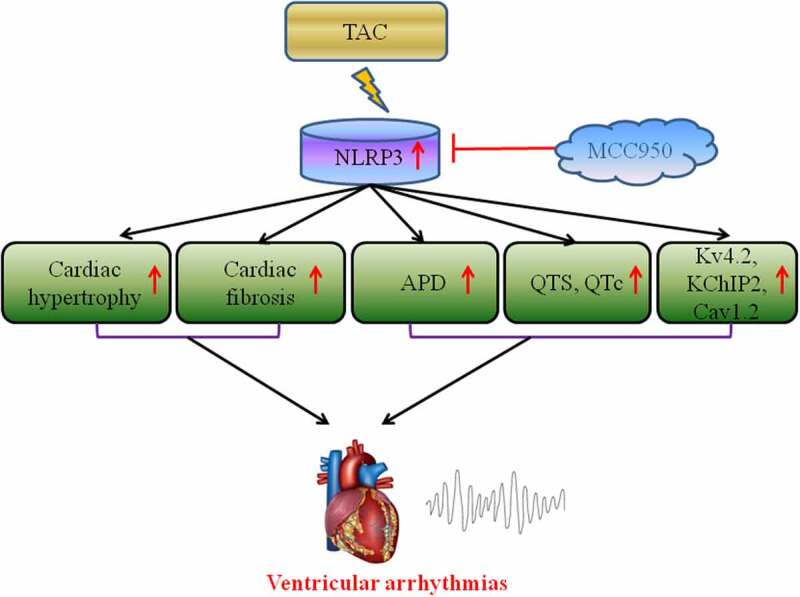

## 1. Introduction

Malignant ventricular arrhythmias (VAs) are the leading cause of sudden cardiac death (SCD), and malignant VAs are the reason for mortality in approximately 50% of heart failure (HF) patients [1,2]. Currently, treatment strategies for VAs following HF remain difficult. Myocardial inflammation may lead to ventricular impairment, progressive HF, and the development of VAs [3,4]. Therefore, it is of considerable significance to inhibit the inflammatory response process for the prevention of VAs after HF.

As it is a widely studied inflammasome, the NLRP3 inflammasome is reported to play a vital role in several inflammatory diseases [5]. A recent review showed that the NLRP3 inflammasome participates in the pathophysiological process of HF by regulating the inflammatory response [6]. NLRP3-IL-1β signaling plays a vital role in the development of VAs after HF [7,8]. Therefore, identifying pharmacological NLRP3 inflammasome inhibitors may be a useful strategy for the prevention and treatment of VAs after HF.

MCC950 is a specific NLRP3 inflammasome inhibitor that has been widely used in animal experiments [9]. MCC950 serves as a cardioprotective agent against myocardial infarction *in vivo* [10,11]. Moreover, MCC950 could attenuate cardiac fibrosis in transverse aortic constriction (TAC)-induced HF mice [12]. Recently, MCC950 was reported to attenuate AF susceptibility in mice [13]. However, the role of MCC950 in the development of HF-induced VAs and the underlying mechanisms remain unclear.

Therefore, the present study aimed to determine the therapeutic potential of targeting the NLRP3 inflammasome with MCC950 in preventing VAs following HF. We hypothesis that MCC950 could decreased the vulnerability to VAs induced by HF by suppressing the activation of the NLRP3 inflammasome.

## 2. Materials and methods

### 2.1 Experimental animals, experimental design, and administration of drugs

A total of 45 male C57BL/6 mice (22–24 g) were obtained from Beijing HFK Bioscience Co., Ltd. The 45 mice were randomly divided into three groups (n = 15/group): the sham group (SHAM), TAC mice with PBS group (HF-PBS), and TAC mice with the MCC950 group (HF-MCC950). MCC950 (10 mg/kg, once per day, i.p.) was administered for 3 weeks following the TAC model induction [10]. The TAC and sham surgeries were performed as described previously [14]. All experimental procedures were according to the guidance of Guidelines for the Care and Use of Laboratory Animals published by the US National Institutes of Health and were approved by the Animal Experiment Center of Fifth People’s Hospital of Chengdu (Approval Number: CDDW2019006).

### 2.2. Echocardiography analysis

Echocardiography analysis was performed as previously reported. Briefly, isoflurane (1.5%) was used to anesthetize the mice, and echocardiography was performed under continuous anesthesia with 1.5% to 2% isoflurane, using a Mylab30CV (ESAOTE) ultrasound system with a 15 Mz probe. Cardiac measurements included the left ventricular end-systolic diameter (LVESD), left ventricular end-diastolic diameter (LVEDD), left ventricular ejection fraction (LVEF), and left ventricular fractional shortening (LVFS) [15].

### 2.3. Surface electrocardiogram, isolated heart preparation, and electrophysiological study

Surface electrocardiogram, isolated heart preparation, and an electrophysiological study were conducted as previously reported [15]. Briefly, mice were anesthetized using 1.5–2% isoflurane. Heart rate and the PR, QRS, and QT intervals were measured. The QT interval was corrected using modified Bazett’s formula. The action potential duration (APD), APD alternans threshold, and VA susceptibility were also measured. VAs were defined as a run of 2 s or more consecutive premature ventricular contractions [15].

### 2.4. Histological analysis

The hearts were embedded in formalin and sliced into 6-μm-thick sections, which were subsequently stained with wheat germ agglutinin (WGA) to quantify cardiac hypertrophy and Masson’s trichrome to quantify cardiac fibrosis [15].

### 2.5. Western blot analysis

Western blotting was performed, as previously described, to evaluate protein expression levels [15]. The primary antibodies used in this study are: NLRP3 (#15101, CST), ASC (#sc-514414, Santa), caspase-1 (#AF5418, Affbiotech), IL-1β (#ab200478, Abcam), IL-18 (#60070-1-Ig, Proteintech Group, Inc.), ANP (#ab225844, Abcam), BNP (#ab236101, Abcam), collagen I (#14695-1-AP, Proteintech Group, Inc.), α-SMA (#ab124964, Abcam), Kv4.2 (#ab123543, Abcam), KChIP2 (#PA5-41075, Invitrogen), Cav1.2 (#ab84814, Abcam), and GAPDH (#ab37168, Abcam). Total protein levels were normalized to those of GAPDH.

### 2.6. Statistical analysis

All data are expressed as the mean ± standard error of the mean (SEM). One-way analysis of variance (ANOVA) was performed using GraphPad Prism software to evaluate differences between the groups. The differences were considered statistically significant at *P* < 0.05.

## 3. Results

In our study, we predicted that MCC950 may reduce ventricular arrhythmias vulnerability induced by heart failure. Therefore, we aimed to investigate the cardio-protective effect of MCC950. Our data revealed that MCC950 could significantly shortened QTc duration, APD_90_, reduced APD alternans threshold and decreased VAs susceptibility following HF, moreover, MCC950 could alleviate the cardiac hypertrophy, fibrosis and reverse the ion remodeling induced by HF. In additional, MCC950 could inhibit the NLRP3-inflammasome by decreasing the protein expression levels of NLRP3, ASC, caspase-1, IL-1β, and IL-18. In summary, our findings demonstrated that MCC950 is expected to be a candidate drug for the therapy of HF-induced VAs.

### 3.1 MCC950 impact on cardiac function in HF mice

**Figure 1.**
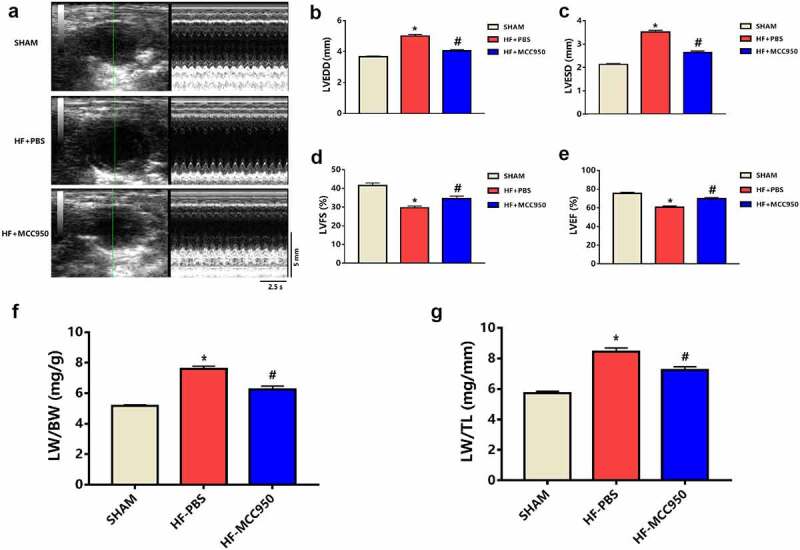
MCC950 impact on cardiac function in HF mice (a) Representative mouse M-mode echocardiograms. (b) LVEDD, (c) LVESD, (d) LVEF, and (e) LVFS values of the mice in each group are presented (n = 6). (f) LW/BW and (g) LW/TL values of the mice in each group are presented (n = 6). Data are expressed as the mean ± SEM. *P < 0.05 vs. sham, ^#^P < 0.05 vs. HF-PBS.

The HF mice exhibited increased LVEDD and LVESD compared to those of the sham mice (*P* < 0.05; Figure 1a–c). Moreover, the HF mice exhibited worse cardiac function, which was indicated by significantly decreased LVFS and LVEF compared with that in the sham group (*P* < 0.05; Figure 1d,e). In addition, the HF mice showed pulmonary edema, indicated by a marked increase in the LW/BW and LW/TL ratios compared to that in the sham group (*P* < 0.05; Figure 1f,g). These effects can be mitigated by treatment with MCC950.

### 3.2 The effect of MCC950 on VAs vulnerabilitys in HF mice

**Figure 2.**
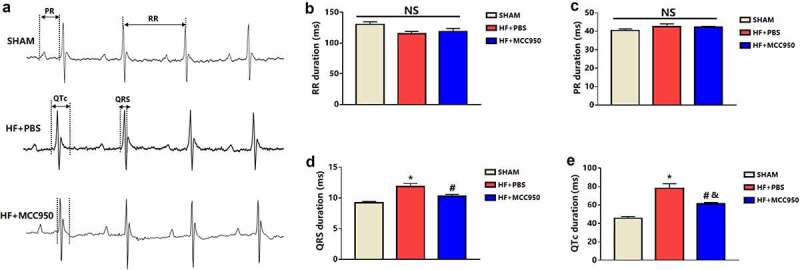
Analysis of surface electrocardiograph in the three groups of mice. (a) A representative trace of surface electrocardiograph for each of the three groups. Surface electrocardiograph parameters of RR duration (b), PR duration (c), QRS duration (d), and QTc duration (e) in the three groups. Data are expressed as the mean ± SEM. *P < 0.05 vs. sham, ^#^ P < 0.05 vs. HF-PBS. NS, not significant, ^&^P < 0.05 vs. sham.

To explore the potential effect of MCC950 on VA susceptibility in HF mice, ECG was conducted in the MCC950-treated mice, and the results were compared with those of the HF group. The results showed that MCC950 administration significantly shortened the QTc duration (Figure 2e). However, MCC950 had no effect on other ECG parameters, including the RR interval, PR interval, and QRS duration (Figure 2b,c).

**Figure 3.**
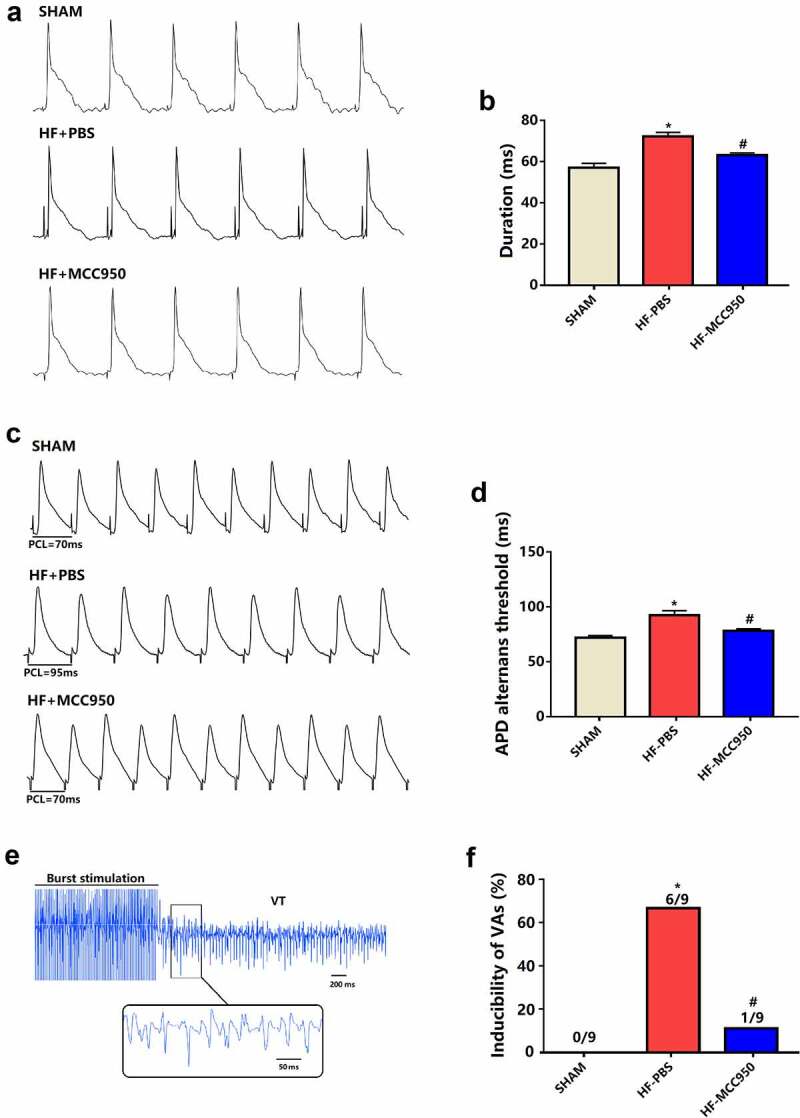
The effect of MCC950 on electrical remodeling and VA susceptibility in HF mice. (a, b) Representative action potential figures and statistical analysis of the APD_90_ (n = 8). (c, d) Representative electric alternans figures and statistical analysis of the ALT thresholds (n = 8). (e, f) Representative arrhythmia induced by burst-pacing stimulations and statistical analysis (n = 9). Data are expressed as the mean ± SEM. *P < 0.05 vs. sham; ^#^P < 0.05 vs. HF-PBS.

Cardiac electrophysiology was subsequently performed. APD_90_ was markedly shortened in the MCC950-treated mice compared with that in sham mice (Figure 3a,b). The threshold of APD alternans was markedly decreased in the MCC950-treated hearts compared with the that in the sham hearts (Figure 3c,d). VAs could not be induced in the sham group (0/9, 0%). The VA induction rate was remarkably increased in the HF group (6/9, 66.7%), and MCC950 administration significantly reduced VA induction compared with that in the HF group (1/9, 11% *vs*. 6/9, 66.7%, Figure 3e,f). These data demonstrate an increased arrhythmic vulnerability in HF mice, and these adverse effects could be mitigated by treatment with MCC950.

### 3.3 The effect of MCC950 on cardiac hypertrophy in HF mice

**Figure 4.**
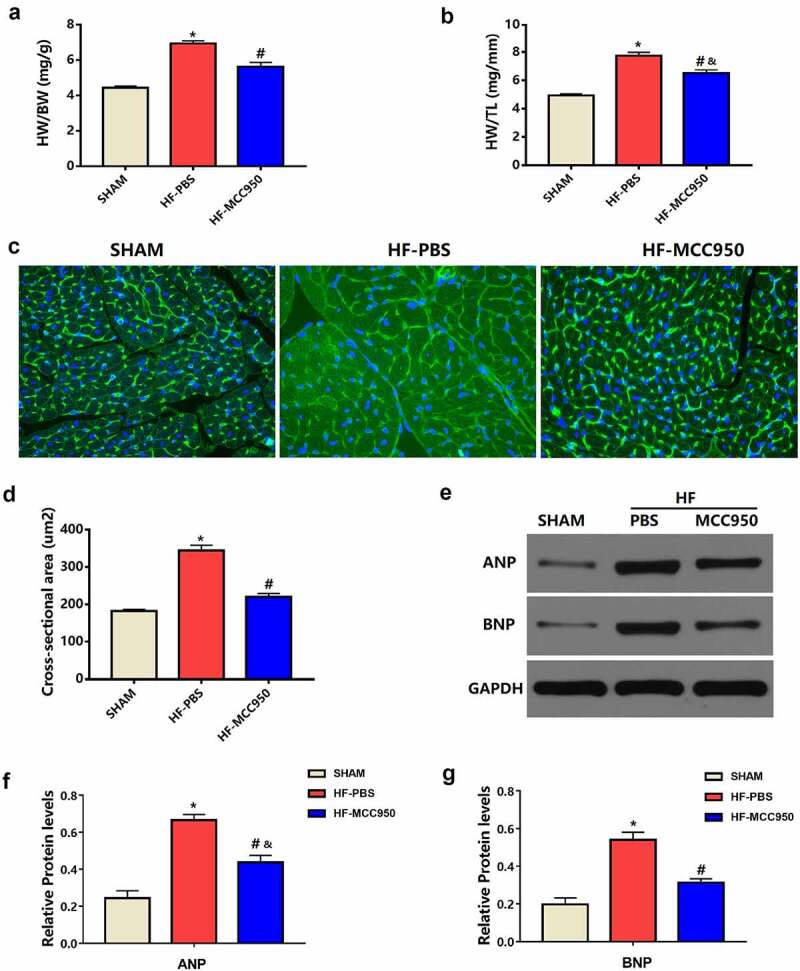
MCC950 impact on myocardial hypertrophy after HF. (a) HW/BW and (b) HW/TL values of the mice in each group (n = 6). (c) Representative WGA staining of left ventricular tissues obtained from mice in each group (magnification, ×400). (d) Statistical analysis of the cardiomyocyte CSA from WGA-stained sections (n = 100+ cardiomyocytes in four samples). (e–g) Representative western blots and statistical analysis of the protein expression levels of the cardiac hypertrophy markers ANP and BNP in each group (n = 4). Data are expressed as the mean ± SEM. *P < 0.05 vs. sham; ^#^P < 0.05 vs. HF-PBS, ^&^P < 0.05 vs. sham.

Left ventricular hypertrophy (LVH) is a powerful independent predictor of VAs [16]. Therefore, we explored the effect of MCC950 on pressure overload-induced HF-related LVH. The HW/BW and HW/TL ratios in the MCC950 mice were markedly decreased compared to those in the HF mice (*P* < 0.05; Figure 4a,b). In addition, WGA staining indicated a significantly increased CSA in the HF-PBS group when compared with that in the sham group, while the CSA was significantly reduced when MCC950 was used (*P* < 0.05; Figure 4c,d). Moreover, the protein expression levels of the LVH markers ANP and BNP were notably elevated in the HF hearts compared to that in the sham hearts (*P* < 0.05; Figure 4e–g), and MCC950 administration markedly reversed HF-induced LVH, as indicated by significantly decreased ANP and BNP protein expression levels (*P* < 0.05; Figure 4e–g). Taken together, these results suggest that MCC950 could reverse cardiac hypertrophy induced by TAC.

### 3.4 MCC950 impact on cardiac fibrosis after HF

**Figure 5.**
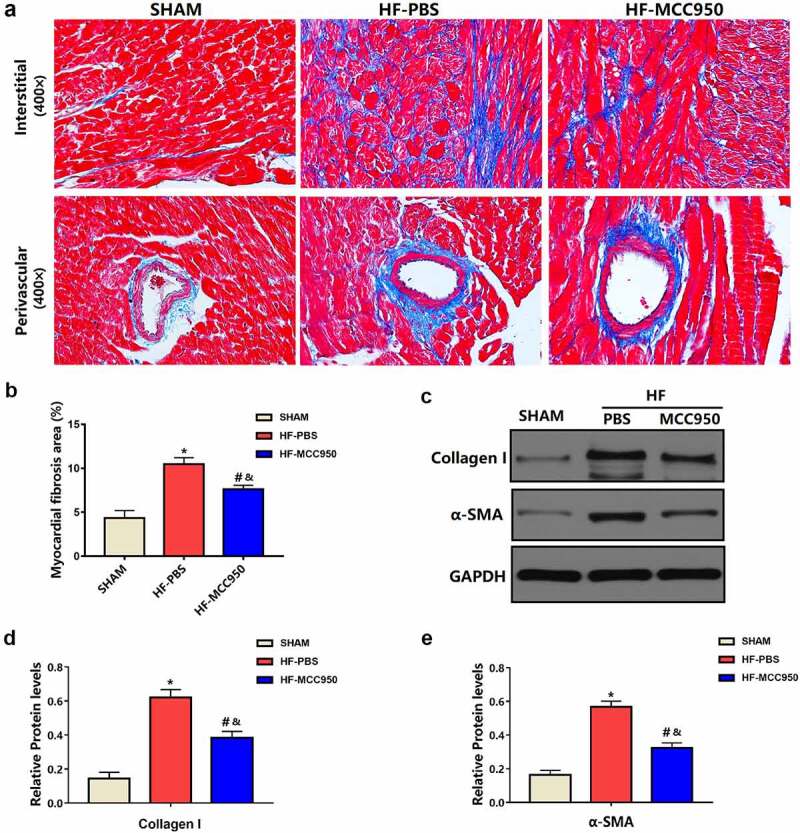
The effect of MCC950 on myocardial fibrosis following HF. (a) Representative Masson staining of myocardial tissues (magnification, ×400). (b) Statistical analysis of the LV collagen volume (%) in Masson-stained sections (n = 6). (c–e) Representative western blots and statistical analysis of the protein expression levels of the cardiac fibrosis markers α-SMA and collagen I in each group. Data are expressed as the mean ± SEM. *P < 0.05 vs. sham; ^#^P < 0.05 vs. HF-PBS, ^&^P < 0.05 vs. sham.

Cardiac fibrosis is a typical pattern shown in the setting of HF, and it promotes VAs by creating a vulnerable substrate for reentrant activity and by favoring the emergence of triggering factors [17]. Therefore, we examined the effect of MCC950 on HF-related cardiac fibrosis. As shown in Figure 5a,b, Masson staining revealed that the LV fibrosis area in MCC950-treated hearts was substantially reduced when compared with that in the HF hearts. Moreover, the protein expression levels of collagen I and α-SMA were substantially decreased in the MCC950-treated mice compared to that in the HF-PBS mice (*P* < 0.05; Figure 5c,e).

### 3.5 MCC950 impact on ion channels remodeling following HF

**Figure 6.**
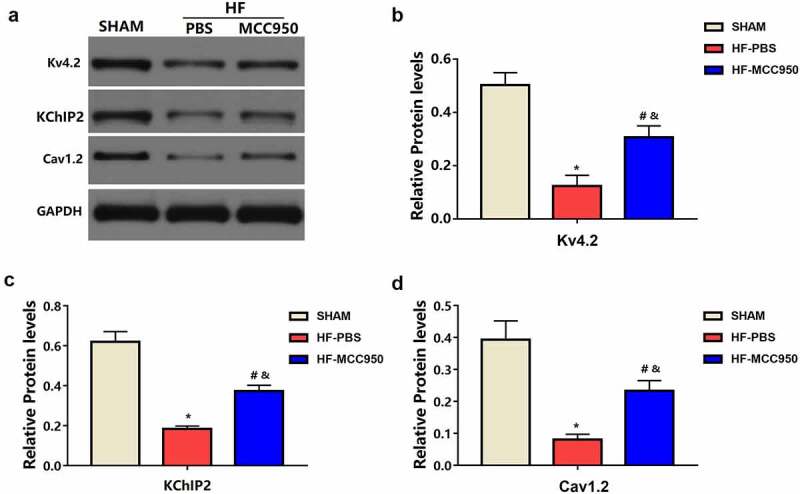
MCC950 impact on the protein expression of ion channels in HF mice. (a–d) Representative western blots and statistical analysis of Kv4.2, KChIP2, and Cav 2.1 in each group. Data are expressed as the mean ± SEM. * P < 0.05 vs. sham; ^#^P < 0.05 vs. HF-PBS, ^&^P < 0.05 vs. sham.

Compared with that in the sham mice, the HF-PBS mice exhibited markedly decreased protein expression of Kv4.2 and KChIP2, which encodes Ito, and Cav1.2, which encodes ICaL (Figure 6a,b). This decreased protein expression was significantly ameliorated in the HF-MCC950 mice. These results indicate that MCC950 treatment significantly upregulated the expression of ion channel proteins (Kv4.2, KChIP2, and Cav1.2) in HF mice.

### 3.6 The effect of MCC950 on NLRP3 inflammasome

**Figure 7.**
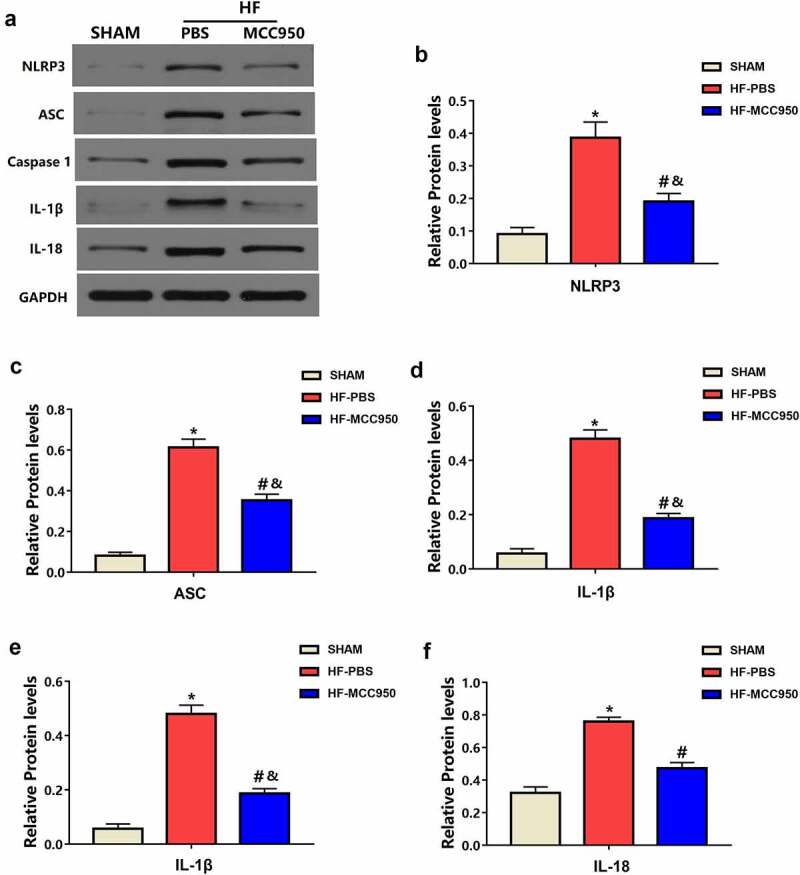
The effect of MCC950 on NLRP3 signaling following HF. Representative western blots (a) and statistical analysis of the protein expression levels of cardiac NLRP3 (b), ASC (c), caspase-1 (d), IL-1β (e), and IL-18 (f) in each group (n = 4). Data are expressed as the mean ± SEM. *P < 0.05 vs. sham; ^#^P < 0.05 vs. HF- HF-PBS, ^&^P < 0.05 vs. sham.

Hypertrophy, fibrosis, and ion channels have essential roles in the mechanisms underlying arrhythmia development after HF [16,17]. NLRP3 deficiency accelerates pressure overload-induced cardiac hypertrophy and fibrosis [18]. Therefore, we further explored the effect of MCC950 on NLRP3 inflammasome signaling in HF mice. Western blotting revealed that TAC-induced HF was associated with increased protein expression of NLRP3, ASC, caspase-1, IL-1β, and IL-18 (Figure 7a–f). However, this effect was attenuated by treatment with MCC950. These results indicate that MCC950 inhibited HF-induced NLRP3 inflammasome activation, causing a decrease in NLRP3, ASC, caspase-1, IL-1β, and IL-18 protein expression.

## 4. Discussion

Previous studies have shown that inhibitors of the NLRP3 inflammasome can reverse the worsening of cardiac function. Wang et al. [19] reported that inhibition of NLRP3 could ameliorate cardiac function in GPER-knockout mice. Furthermore, NLRP3 inhibition ameliorated cardiac dysfunction induced by myocardial infarction [18]. These results are similar to those reported by Birnbaum et al. [20], who reported that targeting the Nlrp3/ASC inflammasome prevents cardiac dysfunction in db/db mice. We found that inhibition of the NLRP3 inflammasome by MCC950 could significantly inhibit pulmonary edema in HF mice.

Clinical evidence indicates that VAs are common in patients with HF [21]. Experimental evidence has shown that activating the NLRP3 inflammasome can markedly increase the risk of VAs after HF [8]. In the current study, we found that MCC950 markedly decreased the susceptibility to pressure overload-induced VAs by inhibiting the NLRP3 inflammasome, as shown by the shortened QTc duration and APD_90_, reduced APD alternans threshold, and lower incidence of VAs. This is in line with previous reports, indicating that diabetes mellitus-induced VAs can be successfully treated by inhibiting the NLRP3 inflammasome [22]. This idea was further supported by experiments showing that NLRP3 deletion could prevent increased VA susceptibility in renal ischemia-reperfusion mice [23]. Therefore, MCC950 may be a potential drug for the prevention and treatment of VAs following HF, which needs to be further confirmed in clinical trials.

Cardiac hypertrophy and fibrosis are essential mechanisms in arrhythmia development after HF [16,17]. Cardiac hypertrophy has been reported to promote reentry and triggered activity, ultimately leading to VAs [16,24]. Inhibition of cardiac hypertrophy can reduce susceptibility to arrhythmias. NLRP3 deficiency aggravates LVH and inflammatory responses in response to pressure overload-induced cardiac remodeling [18]. The current study revealed that MCC950 administration markedly reversed pressure overload-induced cardiac hypertrophy, as indicated by decreased CSA, ANP, and BNP protein expression compared to that in the HF group. Therefore, the present study demonstrated that MCC950 might decrease susceptibility to VAs by reversing HF-induced cardiac hypertrophy.

Cardiac fibrosis is widely accepted to boost VAs by promoting a vulnerable substrate for reentrance [17]. NLRP3 inflammasome blockade could notably reverse fibrosis, including that in the liver and lungs [25,26]. The NLRP3 inflammasome inhibitor MCC950 has also been reported to inhibit cardiac fibrosis induced by MI [10]. In line with previous reports, in the present study, compared to that in the HF group, the MCC950 group showed markedly decreased cardiac fibrosis. Furthermore, MCC950 was reported to reduce cardiac fibrosis *in vitro* [27]. Altogether, MCC950 significantly decreased VA susceptibility by reducing cardiac fibrosis induced by HF.

In addition, electrical remodeling plays an important role in VA pathophysiology. Gomez-Hurtado et al. demonstrated decreased Kv4.2 and KChIP2 protein expression in hearts with TAC-induced HF [28]. Furthermore, protein expression of Cav1.2 was significantly reduced in hearts with TAC-induced HF [29]. In the present study, there was decreased protein expression of Kv4.2, KChIP2, and Cav1.2 in HF hearts compared to that in the sham hearts. Furthermore, we found that inhibiting NLRP3 inflammasome activation could increase the protein expression of Kv4.2, KChIP2, and Cav1.2, which could improve cardiac electrical remodeling and thus reduce susceptibility to VAs after HF.

The NLRP3 inflammasome has been reported to contribute to inflammatory responses in response to adverse cardiac remodeling [12^,30–32^]. Experimental data suggest that IL-1β could promote VAs under pathological conditions, such as renal I/R or DM [22,23]. This notion supports our results, in which the MCC950 group showed markedly reduced cardiac protein expression of NLRP3, ASC, caspase-1, IL-1β, and IL-18. Therefore, MCC950 could significantly decrease susceptibility to VAs by reducing the myocardial inflammatory response, cardiac hypertrophy, and fibrosis by inhibiting NLRP3 inflammasome activation.

There were several limitations to our study. First, There is a lack of data provided regarding in vivo ventricular arrhythmia burden (telemetry) and susceptibility (provocation studies). Moreover, exclusion of female animals from the study has not been justified by the authors and limits its interpretation. Future studies are warranted to validate these findings.

## 5. Conclusion

MCC950 could significantly decrease pressure overload-induced VA vulnerability by reversing HF-induced cardiac hypertrophy, fibrosis, electrical remodeling, and ion channel remodeling via inhibition of the NLRP3 inflammasome. MCC950 is expected to be a candidate drug for the treatment of HF-induced VAs.

## Highlights

- MCC950 reversed HF-induced electrical remodeling.
- MCC950 ameliorated HF-induced cardiac hypertrophy and fibrosis.
- MCC950 could reversing the ion remodeling.
- As a specific NLRP3 inhibitor, MCC950 could reduce HF-induced VAs vulnerability via inhibiting.
